# Prognostic value of right ventricular dysfunction in aortic regurgitation after transcatheter aortic valve replacement

**DOI:** 10.3389/fcvm.2024.1424116

**Published:** 2024-08-30

**Authors:** Yu Mao, Yang Liu, Mengen Zhai, Ping Jin, Haibo Zhang, Lai Wei, Xiaoke Shang, Yingqiang Guo, Xiangbin Pan, Jian Yang

**Affiliations:** ^1^ Department of Cardiovascular Surgery, Xijing Hospital, Xi’an, Shaanxi, China; ^2^ Department of Cardiovascular Surgery, Anzhen Hospital, Capital Medical University, Beijing, China; ^3^ Department of Cardiovascular Surgery, Shanghai Cardiovascular Institution and Zhongshan Hospital, Fudan University, Shanghai, China; ^4^ Department of Cardiovascular Surgery, Union Hospital, Tongji Medical College, Huazhong University of Science and Technology, Wuhan, Hubei, China; ^5^ Department of Cardiovascular Surgery, West China Hospital, Sichuan University, Chengdu, Sichuan, China; ^6^ Department of Cardiology, Fuwai Hospital, National Center for Cardiovascular Disease, Chinese Academy of Medical Science and Peking Union Medical College, Beijing, China

**Keywords:** aortic regurgitation, transcatheter aortic valve replacement, right ventricular dysfunction, prognosis, aortic valve

## Abstract

**Background:**

Aortic regurgitation (AR) may lead to right ventricular dysfunction (RVD), but the prognostic value of RVD in patients undergoing transcatheter aortic valve replacement (TAVR) remains unclear. Our goal was to evaluate the clinical implications, predictors and prognostic significance of RVD in patients with pure AR after TAVR.

**Methods:**

In this multicentre prospective study, patients undergoing TAVR were included between January 2019 and April 2021. The patients were divided into four groups according to the results of transthoracic echocardiography pre- and post-TAVR. The primary end point was 2-year all-cause mortality.

**Results:**

A total of 648 patients were divided into four groups: 325 patients (54.3%) in the no RVD group; 106 patients (17.7%) in the new-onset RVD group; 73 patients (12.2%) in the normalized RVD group; and 94 patients (15.7%) in the residual RVD group. At the 2-year follow-up, there were significant differences in all-cause mortality among the four groups (5.2%, 12.3%, 11.0% and 17.0%, respectively; *p* < 0.05). New-onset RVD was correlated with an increased risk of all-cause death and a composite end point and normalized RVD improved clinical outcomes of baseline RVD. Predictors of new-onset RVD included a higher Society of Thoracic Surgeons score, larger left ventricular end-diastolic volume, lower left ventricular ejection fraction, higher systolic pulmonary artery pressure and smaller RV base diameter.

**Conclusions:**

Changes in periprocedural RVD status significantly affect the risk stratification outcomes after TAVR. Therefore, they may be used as part of decision-making and risk assessment strategies.

**Clinical Trial Registration:**

ClinicalTrials.gov Protocol Registration System (NCT02917980).

## Introduction

Aortic regurgitation (AR) is a common valvular disease, the prevalence of which increases with age and is >2% in people aged >70 years (1). At present, the American College of Cardiology/American Heart Association and the European Society of Cardiology guidelines still recommend surgical aortic valve replacement as the preferred treatment for AR alone (2, 3). However, due to advanced age and severe comorbidities, about 10% of patients with AR cannot undergo operations, and only 20% of them with 30%–50% of left ventricular ejection fraction (LVEF) receive surgical treatment (4).

In recent years, transcatheter aortic valve replacement (TAVR) has led to significant progress in the treatment of severe aortic stenosis (5, 6). Although some studies have preliminarily confirmed that TAVR can achieve significant outcomes in the treatment of pure AR, the optimal timing is still unclear (7). At present, the indications for patients with AR for TAVR are based mainly on symptoms, on left ventricular function and on the degree of dilation observed using echocardiography (2, 3). Therefore, identifying characteristics that predict adverse events is important for risk stratification and may help guide management decisions. Previously, it was recognized that right ventricular dysfunction (RVD) is correlated with adverse events of valvular heart disease that occur postoperatively (8, 9). Long-term AR may lead to chronic pressure and increased volume load, which in turn induces RVD (8). However, understanding of the influence of RVD on the clinical outcomes of TAVR in patients with pure AR is limited (7). The changes in right ventricular (RV) function pre- and post-TAVR have not been explored. In this context, the purpose of this study was to evaluate the clinical implications and prognostic value of RVD after TAVR in patients with pure AR.

## Material and methods

### Study population and design

**Figure 1:**
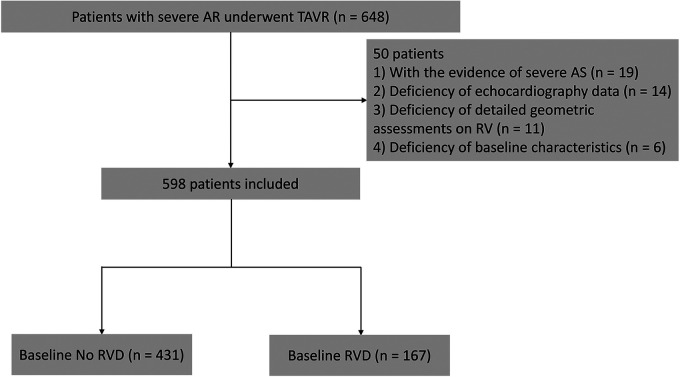
Flow chart of patients with severe aortic regurgitation who underwent transcatheter aortic valve replacement at 6 institutions from January 2019 to April 2021. Patients were divided into two groups on the basis of tricuspid annular plane systolic excursion. AR, aortic regurgitation; AS, aortic stenosis; RV, right ventricle; RVD, right ventricular dysfunction; TAPSE, tricuspid annular plane systolic excursion; TAVR, transcatheter aortic valve replacement.

This study prospectively analysed 648 consecutive patients with severe AR who underwent TAVR at 6 institutions (Xijing Hospital, Beijing Fuwai Hospital, Beijing Anzhen Hospital, Zhongshan Hospital Affiliated with Fudan University, Union Hospital Affiliated with Tongji Medical College of Huazhong University of Science and Technology and West China Hospital) from January 2019 to April 2021. To perform a retrospective analysis of the prospectively collected echocardiographic data, we considered all patients with available transthoracic echocardiograms within 3 months prior to TAVR and at discharge after the procedures. A combination of qualitative and quantitative measurements was used to determine the presence of moderate or severe AR: Patients with moderate or severe AR were qualitatively identified using text that was extracted from the body and the conclusions of each report. Aortic valve (AV) pressure half-time <500 m/s (according to the guidelines of the American Society of Echocardiography) is the primary quantitative measure (10). The exclusion criteria were as follows: (i) The patient had severe aortic stenosis (mean AV pressure gradient >40 mmHg and/or peak flow velocity >4 m/s and/or annular area <1 cm^2^) (*n* = 19) (11); (ii) the patient's echocardiogram was missing (*n* = 14) or the image was not focused on the right ventricle (RV) for detailed geometric assessments (*n* = 11); or (iii) patient information related to baseline characteristics (*n* = 6) was missing (Figure 1). The mid-term outcomes of the four groups were compared according to preprocedural RV function status: no RVD group (no RVD before and after TAVR), new-onset RVD group (no RVD before but RVD after TAVR), normalized RVD group (RVD before but not after TAVR) and residual RVD group (RVD before and after TAVR). This study complied with the Declaration of Helsinki and was approved by the ethics committee of each participating hospital. All patients provided written informed consent for the procedure and for subsequent data collections.

### Evaluation of RV function

Transthoracic echocardiography was conducted by experienced sonographers using the Philips iE 33 machine (Philips Healthcare, Andover, MA, USA) and systematically reviewed by experienced experts. According to current recommendations, evaluations included M-mode, two-dimensional and colour, continuous and pulsed-wave Doppler echocardiography (12). The echo loop was analysed at the workstation that allowed offline analysis (Syngo Dynamics Workplace, Version 9.5, Siemens Medical Solutions, Malvern, PA, USA).

According to the current guidelines of the American Society of Echocardiography/European Association of Cardiovascular Imaging (13), and the statements of the Heart Failure Association and the Working Group on Pulmonary Circulation and Right Ventricular Function of European Society of Cardiology (14), the presence of RVD was predefined by the following RV function and size parameters: fraction area change (FAC) [(RV end-diastolic area—RV end-systolic area)]/RV end-diastolic area × 100%), tricuspid annular plane systolic excursion (TAPSE) and right ventricular lateral systolic motion using tissue Doppler imaging (S’). The critical values for RVD are as follows: FAC < 35%, TAPSE < 1.7 cm and S’ < 9.5 cm/s. If >50% of the available RV function parameters are below the lower threshold, RVD exists. The hierarchical evaluation of RV function parameters was repeated. The main classification was TAPSE < 1.7 cm; if TAPSE was not available, the classification was S’ < 9.5 cm/s. If neither TAPSE nor S’ is available, use FAC < 35% to determine RV function status.

### Procedures

Under local anaesthesia and conscious sedation, all patients underwent TAVR via the transapical approach using the J-Valve (Jiecheng Medical Co., LTD., Suzhou, China). The procedures were previously described in detail (15). The size of the transcatheter heart valve was determined by the individual heart centres based on preprocedural transthoracic echocardiography and multidetector computed tomography. Postprocedural care included heart rate monitoring for at least 48 h, laboratory tests and a 12-lead electrocardiogram immediately after the procedure, followed by daily echocardiographic monitoring prior to discharge.

### Follow-up and end points

After discharge, follow-up was carried out at 30 days, 1 year and 2 years after TAVR. Follow-up with the patient was conducted through clinical visits and/or telephone contact after a predetermined time point.

The primary end point was 2-year all-cause mortality. Secondary end points included a combination of major adverse cardiovascular and cerebrovascular events (all-cause mortality, myocardial infarction and stroke) and rehospitalization for heart failure at 2 years. Hospitalization for heart failure is defined as the presence of symptoms or signs of heart failure and the use of diuretics during hospitalization. Procedural and other related complications during TAVR [including life-threatening bleeding, acute kidney injury (≥ stage 3) and severe puncture site complications] were evaluated according to Valvular Academic Research Consortium-3 criteria (16).

### Statistical analysis

All data were tested for normality and homogeneity of variance. Continuous variables were expressed as mean ± standard deviation or median and interquartile ranges (IQR). The results of the categorical data were expressed as *n* (%). Patients were classified according to RV function status. Normal distribution of continuous and non-normal variables was compared using Bonferroni's analysis of variance test and the Mann–Whitney *U* test, respectively, and the *χ*^2^ test or the Fisher exact test, if appropriate. Univariate and multivariate logistic regression models (entered when the univariate *p* value < 0.05) was used to derive factors associated with residual RVD and new-onset RVD. We used pre-TAVR RVD as a binary variable and pre-TAVR FAC, TAPSE and S’ as continuous variables, respectively. Residual RVD was compared with normalized RVD, whereas new-onset RVD was compared with no RVD. Cox regression models were used to derive the hazard ratio (HR) (entered when the univariate *p* value < 0.05) and 95% confidence intervals (CI) for clinical, laboratory and echocardiographic factors associated with adverse events. The HRs were calculated in univariate and multivariate Cox regressions to assess the correlations of FAC, TAPSE and S’ with study outcomes. In addition to guideline-based procedural indications as a decision tool, decision curve analysis was used to estimate the net benefit of RV evaluation (17). Kaplan-Meier curves were used to determine the 2-year cumulative incidence of all-cause mortality and the composite end point. Bilateral *p* values < 0.05 were considered statistically significant. Statistical analyses were performed using R programming language version 4.2.2 (R Foundation for Statistical Computing, Vienna, Austria).

## Results

### Baseline characteristics

**Figure 2:**
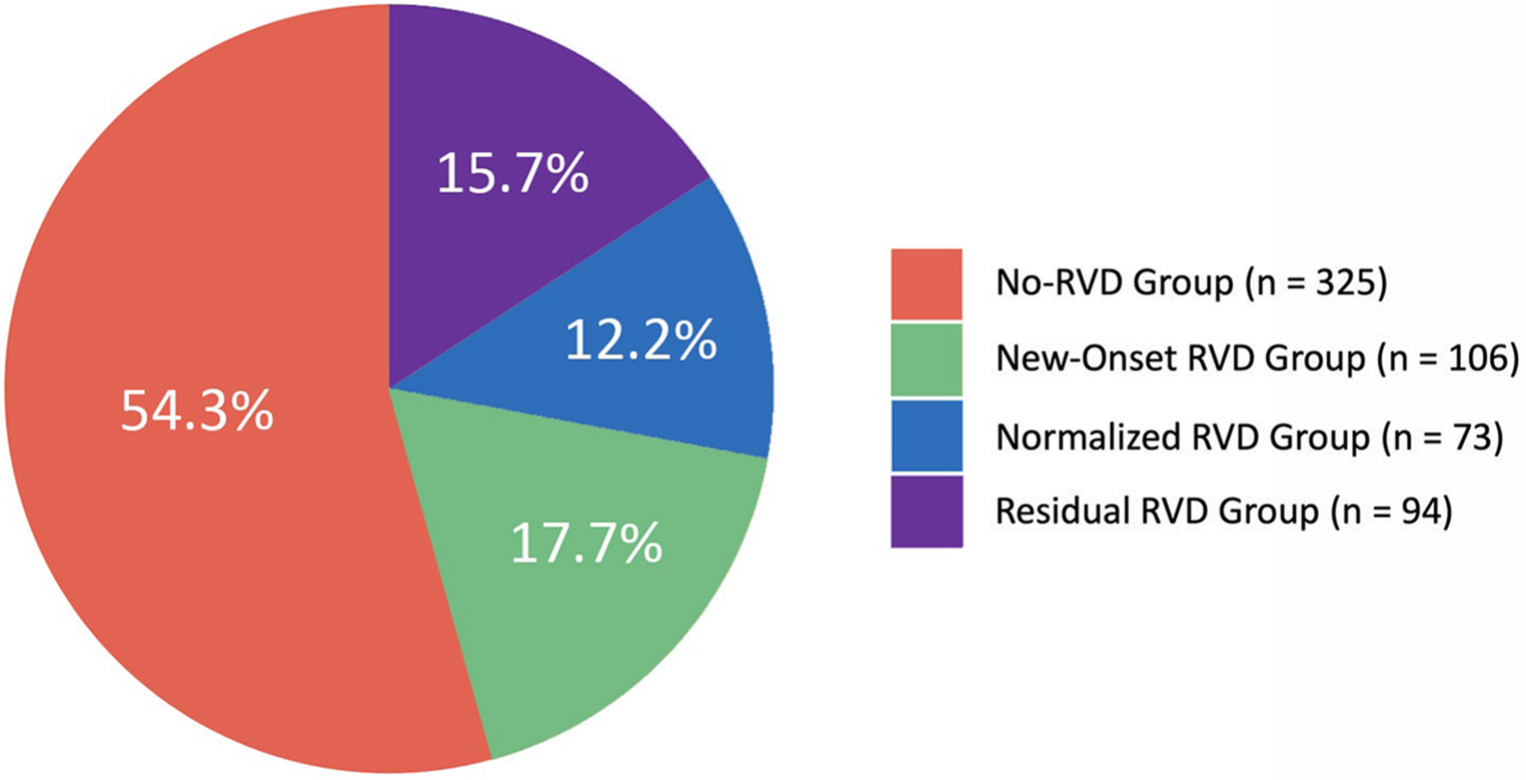
Distribution of periprocedural RVD changes in patients undergoing TAVR. RVD, right ventricular dysfunction; TAVR, transcatheter aortic valve replacement.

The average age of patients in the overall cohort was 72.0 (IQR: 66.0–78.0) years; 75.1% were male, with a Society of Thoracic Surgeons (STS) score of 6.8 (4.1–8.8)% and European System for Cardiac Operative Risk Evaluation (EuroSCORE) II of 6.2 (3.5–8.3)%. Patients were divided into four groups according to RV functional status, as shown below: 325 patients (54.3%) in the no RVD group, 106 patients (17.7%) in the new-onset RVD group, 73 patients (12.2%) in the normalized RVD group and 94 patients (15.7%) in the residual RVD group (Figure 2). The baseline characteristics of each group are shown in Table 1. Compared with the no-baseline RVD group, patients in the baseline RVD group had more males (82.6% vs. 72.2%, *p* < 0.001) with a higher prevalence of diabetes, hypertension, hyperlipidaemia, coronary artery disease and chronic obstructive pulmonary disease. In particular, the prevalence of diabetes, hypertension and hyperlipidaemia was higher in the residual RVD group. As expected, the STS score and the EuroSCORE II in the baseline RVD group were higher than those in the no-baseline RVD group.

**Table 1:** Baseline characteristics.

	Baseline no RVD		Baseline RVD		*p*-value
	No RVD (*n* = 325)	New-onset RVD (*n* = 106)	Normalized RVD (*n* = 73)	Residual RVD (*n* = 94)	All groups
Age, years	72.0 (65.0–77.0)	72.5 (67.0–79.0)	71.0 (66.0–80.0)	73.0 (66.0–80.0)	0.256
Female	30.2 (98)	20.8 (22)	15.1 (11)	19.1 (18)	**0** **.** **011**
Body mass index, kg/m^2^	23.4 (20.3–26.6)	22.1 (19.9–24.7)	22.0 (19.6–24.6)	22.3 (19.0–24.7)	**<0.001**
Body surface area, m^2^	1.6 (1.3–1.8)	1.5 (1.3–1.7)	1.5 (1.4–1.6)	1.6 (1.3–1.7)	0.059
NYHA functional class III or IV	95.4 (310)	98.1 (104)	98.6 (72)	98.9 (93)	0.262
Diabetes mellitus	20.9 (68)	18.9 (20)	24.7 (18)	36.2 (34)	**0**.**012**
Hypertension	60.0 (195)	67.0 (71)	68.5 (50)	79.8 (75)	**0**.**004**
Hypercholesterolaemia	24.3 (79)	25.5 (27)	27.4 (20)	39.4 (37)	**0**.**036**
Coronary artery disease	28.3 (92)	30.2 (32)	26.0 (19)	42.6 (40)	0.050
Percutaneous coronary intervention	3.1 (10)	2.8 (3)	5.5 (4)	5.3 (5)	0.525
Coronary artery bypass surgery	3.4 (11)	1.9 (2)	4.1 (3)	5.3 (5)	0585
Stroke or TIA	3.4 (11)	3.8 (4)	5.5 (4)	2.1 (2)	0.681
Peripheral vascular disease	40.3 (131)	38.7 (41)	41.1 (30)	43.6 (41)	0.911
Chronic obstructive pulmonary disease	8.0 (26)	8.5 (9)	13.7 (10)	14.9 (14)	0.146
Dialysis	23.7 (77)	17.9 (19)	21.9 (16)	23.4 (22)	0.660
Atrial ﬁbrillation	20.9 (68)	26.4 (28)	24.7 (18)	28.7 (27)	0.366
Permanent pacemaker implant	3.4 (11)	5.7 (6)	1.4 (1)	6.4 (6)	0.289
STS score,%	6.7 (3.2–9.0)	6.2 (4.7–7.9)	7.1 (5.5–8.3)	7.7 (5.9–9.1)	0.083
EuroSCORE II,%	6.0 (2.9–8.5)	6.2 (4.0–7.8)	6.2 (5.5–7.9)	7.4 (6.0–8.6)	**0**.**021**

EuroSCORE II, European system for cardiac operative risk evaluation II; NYHA, New York Heart Association; RVD, right ventricular dysfunction; STS, society of thoracic surgeons; TIA, transient ischemic attack.

The bold *p* value means that the value is <0.05.

The echocardiographic data of each group are summarized in Table 2. Compared with the no-baseline RVD group, the left ventricular end-diastolic volume (LVEDV), right ventricle–right atrial pressure gradient and the peak flow velocity of tricuspid regurgitation were higher in the baseline RVD group; the left ventricular ejection fraction, mean AV pressure gradient and AV pressure for half-time were lower. As expected, the prevalence of ≥moderate mitral regurgitation and ≥moderate tricuspid regurgitation was higher in baseline RVD patients, whereas the FAC and TAPSE values were lower.

**Table 2:** Echocardiographic characteristics.

	Baseline no RVD		Baseline RVD		*p* value
	No RVD (*n* = 325)	New-onset RVD (*n* = 106)	Normalized RVD (*n* = 73)	Residual RVD (*n* = 94)	All groups
Left ventricular ejection fraction, %	54.0 (50.0–58.0)	49.0 (45.0–55.0)	42.0 (37.0–49.0)	43.5 (38.0–51.0)	**<0.001**
Aortic valve area, cm^2^	0.6 (0.5–0.7)	0.5 (0.4–0.6)	0.6 (0.4–0.6)	0.5 (0.4–0.6)	**<0.001**
Peak velocity, m/s	1.8 (1.6–2.1)	1.9 (1.7–2.1)	2.2 (1.9–2.5)	2.1 (1.9–2.4)	**<0.001**
Mean gradient, mmHg	7.2 (4.6–10.2)	6.3 (4.8–7.8)	3.8 (1.5–7.1)	4.9 (2.6–6.7)	**<0.001**
AV pressure for one-half time	410.0 (362.0–465.0)	408.5 (347.0–465.0)	357.0 (314.0–420.0)	378.0 (335.0–411.0)	**<0.001**
E/e’	2.1 (2.0–2.2)	2.2 (2.0–2.3)	2.2 (2.1–2.4)	2.2 (2.0–2.4)	**<0.001**
LVEDV, ml/m^2^	52.1 (43.6–60.6)	56.4 (46.7–64.7)	60.2 (46.6–68.2)	56.6 (43.4–66.8)	**<0.001**
LVESV, ml/m^2^	22.8 (17.6–29.0)	28.6 (21.9–34.4)	27.6 (23.5–31.0)	27.3 (23.7–30.6)	**<0.001**
Left atrial volume index, ml/m^2^	42.4 (34.3–52.1)	51.1 (38.9–61.1)	51.4 (36.8–59.9)	47.5 (33.7–58.8)	**<0.001**
Mitral regurgitation ≥ moderate	20.6 (67)	29.2 (31)	41.1 (30)	31.9 (30)	**0.001**
Tricuspid regurgitation ≥ moderate	12.3 (40)	35.0 (37)	41.1 (30)	28.7 (27)	**<0.001**
FAC, %	42.1 (37.8–45.9)	36.2 (31.3–39.6)	31.1 (27.6–34.1)	31.1 (27.5–35.3)	**<0.001**
DTI (S’), cm/s	10.9 (8.8–13.2)	9.6 (7.2–12.3)	10.0 (6.9–12.5)	9.6 (7.0–12.2)	**<0.001**
TAPSE, cm	2.0 (1.7–2.4)	1.8 (1.6–1.9)	1.3 (0.9–1.6)	1.3 (1.0–1.7)	**<0.001**
Systolic pulmonary artery pressure, mmHg	37.0 (30.7–45.0)	53.9 (47.0–60.5)	46.8 (42.9–54.4)	44.8 (40.9–51.7)	**<0.001**
RV–RA gradient, mmHg	38.1 (33.5–43.8)	42.9 (37.4–47.1)	44.6 (38.5–50.1)	43.6 (35.8–49.1)	**<0.001**
TR peak velocity, m/s	2.7 ± 0.6	3.2 ± 0.5	3.7 ± 0.6	3.6 ± 0.7	**<0.001**
RV basal diameter, mm	36.9 (31.9–42.4)	29.7 (25.8–33.2)	36.0 (32.8–42.7)	37.3 (32.1–44.3)	**<0.001**
RV mid-cavity diameter, mm	18.2 (15.9–21.5)	18.4 (15.8–21.5)	19.3 (16.8–22.0)	19.7 (17.1–22.2)	**0.048**

AV, aortic valve; DTI, diffusion tensor imaging; FAC, fraction area change; LVEDV, left ventricular end-diastolic volume; LVESV, left ventricular end-systolic volume; RA, right atrium; RV, right ventricle; RVD, right ventricular dysfunction; S’, right ventricular lateral systolic motion using tissue doppler imaging; TAPSE, tricuspid annular plane systolic excursion; TR, tricuspid regurgitation.

The bold *p* value means that the value is <0.05.

### Procedural details and hospitalization outcomes

Procedural details and hospitalization outcomes are shown in Table 3. The procedural time of the overall cohort was 109.0 (IQR: 83.0–144.0) min; fluoroscopy time was 8.2 (IQR: 6.1–11.2) min; and contrast medium volume was 622.0 (IQR: 561.0–695.0) mGy. There were no significant differences in procedural details among the four groups.

**Table 3:** Procedural and in-hospital details.

	Baseline no RVD		Baseline RVD		*p*-value
	No RVD (*n* = 325)	New-onset RVD (*n* = 106)	Normalized RVD (*n* = 73)	Residual RVD (*n* = 94)	All groups
Procedural details					
Procedure time, min	112.0 (81.0–147.0)	107.0 (85.0–143.0)	102.0 (84.0–133.0)	107.0 (84.0–151.0)	0.623
Fluoroscopy time, min	8.4 (5.9–11.2)	8.1 (6.2–11.3)	7.7 (6.2–10.5)	8.2 (6.2–12.1)	0.863
Contrast medium volume, ml	627.0 (572.0–686.0)	616.0 (554.0–711.0)	600.0 (554.0–686.0)	616.0 (554.0–736.0)	0.527
Device success	96.9 (315)	93.4 (99)	94.5 (69)	95.7 (90)	0.344
Conversion to an operation	1.2 (4)	2.8 (3)	4.1 (3)	2.1 (2)	0.259
Coronary obstruction	0.6 (2)	2.8 (3)	1.4 (1)	0 (0)	0.127
Aortic root injury	1.5 (5)	1.9 (2)	1.4 (1)	2.1 (2)	0.925
Valve-in-valve	0.6 (2)	0 (0)	0 (0)	0 (0)	1.000
New permanent pacemaker	9.5 (31)	10.4 (11)	4.1 (3)	7.4 (7)	0.426
In-hospital details					
Myocardial infarction	0.3 (1)	1.0 (1)	1.4 (1)	1.1 (1)	0.267
Stroke	0.9 (3)	0 (0)	0 (0)	0 (0)	1.000
Life-threatening or major bleeding	0 (0)	1.0 (1)	1.4 (1)	1.1 (1)	0.091
Major vascular complication	0.9 (3)	1.0 (1)	0 (0)	1.1 (1)	1.000
Pacemaker implanted in-hospital	6.2 (20)	11.8 (12)	5.7 (4)	16.3 (15)	**0**.**011**
Acute kidney injury	7.8 (25)	6.9 (7)	10.0 (7)	14.1 (13)	0.235
Duration between procedures and TTE reassessment	5.5 (3.0–7.0)	6.0 (4.0–7.0)	7.0 (5.0–9.0)	6.5 (4.0–8.0)	0.418
Aortic regurgitation ≥ moderate	0.9 (3)	0 (0)	2.9 (2)	1.1 (1)	0.273
Mitral regurgitation ≥ moderate	19.3 (62)	28.4 (29)	37.1 (26)	31.5 (29)	**0**.**003**
Length of hospital stay, days	10.0 (7.0–13.0)	10.0 (7.0–12.0)	14.5 (8.0–19.0)	13.5 (8.0–19.0)	**<0.001**

RVD, right ventricular dysfunction; TTE, transthoracic echocardiography.

The bold *p* value means that the value is <0.05.

There were no significant differences in the incidence of major adverse events (such as myocardial infarction, stroke, life-threatening bleeding, major vascular complications and acute kidney injury). Notably, the incidence of implanting a new permanent pacemaker during hospitalization was higher in the new-onset RVD group and the residual RVD group. Compared with the no RVD group, a higher proportion of patients in the other three groups had ≥moderate MR on transthoracic echocardiography measurements before discharge. In addition, hospital stays were longer in the baseline RVD group than in the no-baseline RVD group [14.0 (8.0–19.0) days vs. 10.0 (7.0–13.0) days, *p* < 0.001].

### Clinical outcomes

**Figure 3:**
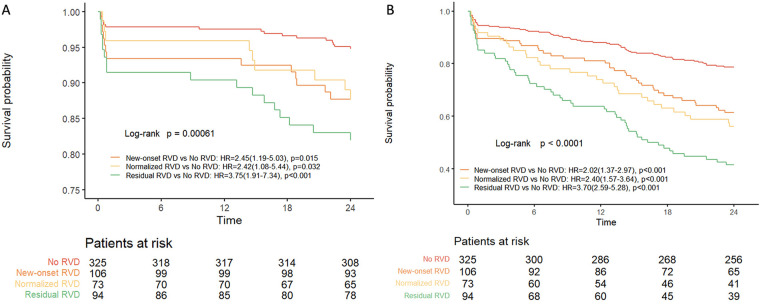
Kaplan–Meier analysis of all-cause death **(A)** and composite end point **(B)** at 2 years according to the presence or absence of RVD. CI, conﬁdence interval; HR, hazard ratio; RVD, right ventricular dysfunction.

Kaplan-Meier curves showed that, compared with the no RVD group, the new-onset RVD group (12.3% vs. 5.2%; HR: 2.45; 95% CI: 1.19–5.03; *p* = 0.015) and the normalized RVD group (11.0% vs. 5.2%; HR: 2.42; 95% CI: 1.08–5.44; *p* = 0.032) and the residual RVD group (17.0% vs. 5.2%; HR: 3.75; 95% CI: 1.91–7.34; *p* < 0.001) had significantly higher all-cause mortality (Figure 3). Importantly, the clinical outcomes of the new-onset RVD group were much worse than those of the no RVD group, whereas the clinical outcomes of the normalized RVD group were comparable with those of the residual RVD group. There were significant differences in the 2-year composite end points (21.2%, 38.7%, 43.8% and 58.5%, respectively, in the no RVD, new-onset RVD, normalized RVD and residual RVD groups (all *p* < 0.001) (Figure 3).

The results of univariate and multivariate Cox regression analysis for 2-year all-cause mortality are shown in Table 4. After multivariate adjustment, the new-onset RVD, normalized RVD and residual RVD were significant predictors of 2-year all-cause mortality compared with the no RVD group. Univariate and multivariate analyses for the 2-year composite end point are displayed in Supplementary Table S1. The new-onset RVD group was the risk factor for the 2-year composite end point (HR: 1.79; 95% CI: 1.01–3.17; *p* = 0.049), whereas being female was the protective factor (HR: 0.55; 95% CI: 0.33–0.95; *p* = 0.030).

**Table 4:** Univariable and multivariable cox regression analyses of 2-year all-cause mortality.

	Univariate analysis		Multivariable analysis	
	HR (95% CI)	*p*-value	HR (95% CI)	*p*-value
Diabetes mellitus	3.263 (1.974, 5.394)	0.008		
Hypercholesterolaemia	2.994 (1.812, 4.946)	0.003		
Stroke or TIA	2.457 (1.327, 3.799)	0.001		
STS score	1.144 (1.065, 1.229)	<0.001		
Left ventricular ejection fraction	0.947 (0.918, 0.978)	0.001		
TR peak velocity	1.775 (1.265, 2.490)	0.046		
New-onset RVD group vs. no RVD group	2.445 (1.188, 5.034)	0.005	3.412 (1.290, 9.025)	0.013
Normalized RVD group vs. no RVD group	4.220 (2.107, 8.450)	<0.001	3.024 (2.397, 4.772)	0.003
Residual RVD group vs. no RVD group	3.519 (1.778, 6.966)	<0.001	2.297 (1.576, 3.690)	0.013
TAPSE	0.553 (0.338, 0.905)	0.018		

RVD, right ventricular dysfunction; STS, society of thoracic surgeons; TAPSE, tricuspid annular plane systolic excursion; TIA, transient ischemic attack; TR, tricuspid regurgitation.

**Figure 4:**
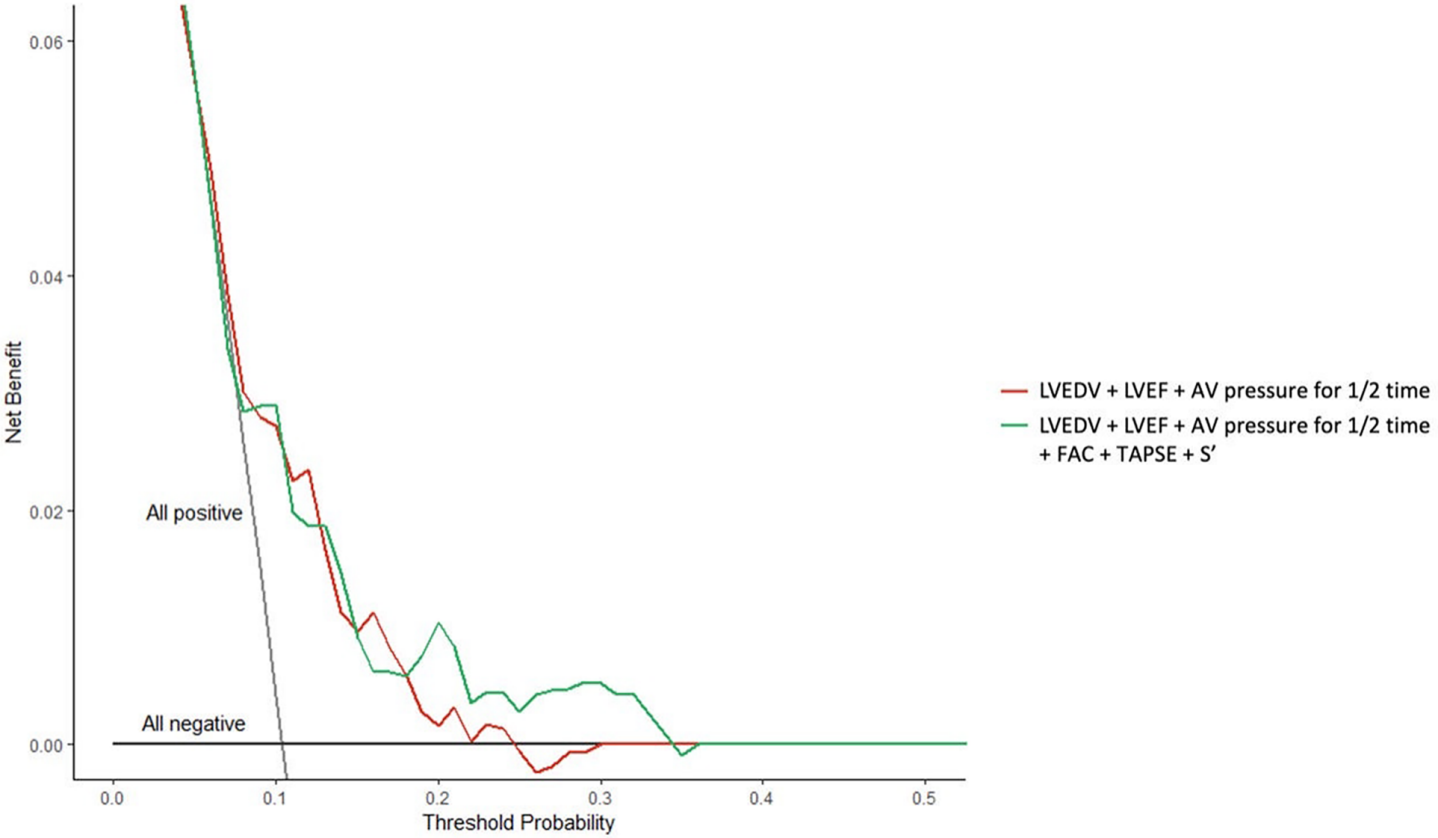
Decision curve analysis of the right ventricular remodelling patterns for risk prediction. AV, aortic valve; FAC, fraction area change; LVEDV, left ventricular end-diastolic volume; LVEF, left ventricular ejection fraction; S’, right ventricular lateral systolic motion using tissue Doppler imaging; TAPSE, tricuspid annular plane systolic excursion.

Compared with traditional risk factors, the RV remodelling model improved risk prediction. Inclusion of RV variables (TAPSE, FAC and S’) provided a consistent positive and larger net clinical benefit compared with the degree of AR (AV pressure for half-time), LV size (LVEDV) and LV function (LVEF) alone (Figure 4).

### RV function changes after TAVR

New-onset RVD group vs. No RVD group. When TAPSE < 1.7 cm was used as a bivariate or continuous variable, multivariate analysis showed that the variables that identified new-onset RVD included higher STS score (*p* = 0.027; *p* = 0.031), larger LVEDV (*p* = 0.003; *p* = 0.004) and lower LVEF (*p* = 0.004; *p* = 0.003), higher systolic pulmonary artery pressure (sPAP) (*p* < 0.001; *p* < 0.001) and smaller RV base diameter (*p* < 0.001; *p* < 0.001) (Supplementary Table S2).

Normalized RVD group vs. Residual RVD group. As shown in Supplementary Table S3 (when TAPSE < 1.7 cm was used as a bivariate or continuous variable), previous coronary artery disease (*p* = 0.023; *p* = 0.029), lower EuroSCORE II (*p* = 0.029; *p* = 0.042) and lower sPAP (*p* = 0.017; *p* = 0.009) were identified as the predictors of normalized RVD.

## Discussion

Our study reported the clinical implications and prognostic value of RVD after TAVR in patients with pure AR. It is the largest data set to date to investigate the prognostic impact of RVD. The main findings were as follows: (i) Baseline RVD was correlated with the adverse events. (ii) Changes in periprocedural RVD (whether new-onset or normalized) significantly affected the risk stratification after TAVR. Among the changes, new-onset RVD was correlated with an increased risk of all-cause death and composite end point, and normalized RVD improved clinical outcomes of baseline RVD. (iii) Predictors of new-onset RVD included higher STS score, larger LVEDV, lower left ventricular ejection fraction, higher sPAP and smaller RV base diameter compared with no RVD. (iv) The predictors of RVD normalization were previous coronary artery disease, lower EuroSCORE II and lower sPAP.

Previous studies on RVD improvement in patients with AR undergoing TAVR were limited. In this cohort, 27.9% of patients had baseline RVD according to the current American Society of Echocardiography/European Association of Cardiovascular Imaging guidelines (13), the statements of the Heart Failure Association and the Working Group on Pulmonary Circulation and Right Ventricular Function of European Society of Cardiology (14) and an outcome comparable to those of previous aortic stenosis studies (18–20). Left ventricular dilation and eventual LV systolic dysfunction are thought to be pathophysiological consequences of remodelling and represent key triggers for valve treatment (21). Aortic regurgitation may cause LV remodelling and dilation, and the increase of volume and pressure load can progressively lead to left atrium, tricuspid valve/pulmonary circulation and eventually RV overload (22). In addition, left ventricular remodelling induced by AR may reduce the contractile force of the interventricular septum, resulting in RVD (23). However, previous studies have demonstrated that RVD can be used to monitor disease progression, periprocedural status and prognostic outcomes in patients with AV disease. Although the guidelines still recommend surgical aortic valve replacement as the preferred treatment for pure AR (2, 3), the risk of RV worsening after the procedure, especially in patients with baseline RVD, may lead the cardiac team to favour interventional therapy (20).

Previous studies evaluated RVD changes in patients with aortic stenosis after TAVR. However, the reported RVD results and clinical outcomes were contradictory (18, 20, 24). Lindman and co-workers showed that baseline RVD was not correlated with an increased risk of death after the procedure (24). Furthermore, Koifman and colleagues analysed the 1-year mortality risk of patients with RVD and also found no statistically significant difference (18). In contrast, Asami and colleagues showed that RVD was robust for a 1-year mortality risk (20). Similarly, several studies have explored the impact of the geometry and function of the right cardiac system on the prognosis of patients with severe AR and have similarly yielded contradictory outcomes (25, 26). In the preceding studies, differences in statistical analytical methods and inconsistent definitions of RVD may be the Achilles’ heel that led to contradictory outcomes. Our findings are the first to date to report clinical outcomes of RVD after TAVR in patients with pure AR. The 2-year all-cause mortality and the incidence of the composite end point were significantly higher in the new-onset RVD group, the normalized RVD group and the residual RVD group compared with the no RVD group. Importantly, consistent with our previous study, we characterized RVD in terms of TAPSE (FAC or S’) (19). After multivariate adjustment, the new-onset RVD group, the normalized RVD group and the residual RVD group were significant predictors of 2-year all-cause mortality compared with the no RVD group. The new-onset RVD group and being female were predictors of the 2-year composite end point. Sensitivity analyses suggested that RV assessments (TAPSE, FAC and S’) may exclude confounding effects of AR or LV morphology or dysfunction, providing a greater net clinical benefit to identify the influence of periprocedural RVD changes.

The main contribution of our study was the strong correlation of the new-onset RVD after TAVR with poorer clinical outcomes. Patients with new-onset RVD had a 2.45-fold increase in 2-year all-cause mortality and a 1.97-fold increase in the composite end point. These results are similar to those of the previous reports (20, 27). Furthermore, a higher STS score, a larger LVEDV, a lower left ventricular ejection fraction, a higher sPAP score and a smaller RV base diameter may lead to new-onset RVD after TAVR.

Of all patients who had baseline RVD, RVD normalized in 43.7% of patients. Similar to the outcomes of previous studies, normalization of RVD reduced the risk of all-cause death (20, 28). However, RV functional recovery was not defined solely as an improvement in RVD severity but was evaluated using multiple parameters. Notably, this study revealed several factors that predicted RVD normalization, including previous coronary heart disease, lower EuroSCORE II and lower sPAP. In particular, the prediction of previous coronary heart disease is novel and has not been reported in previous studies.

In current guidelines, the presence of RVD is not an indication of TAVR for patients with severe AR (2, 3, 29). Although we did not report an exciting therapeutic effect in the current paper, the results of this study demonstrated that even mild sPAP was correlated with an increased risk. Moreover, the reason we have not yet found a difference between normalized and residual RVD may be attributed to limited follow-up time. Therefore, further research is needed to understand any possible benefits of early TAVR for AR in the presence of RVD. In addition, only 20% of patients with severe AR receive surgical treatment due to advanced age or comorbidities (4), whereas treatment of other patients is often delayed due to poor risk assessment. Importantly, 75.4% of patients in this study had no RVD after the procedure, whereas nearly half of the patients (43.7%) had normalized RVD, which is an important outcome. Finally, based on our findings, TAPSE, FAC and/or S’ may serve as practical markers of TAVR risk stratification in patients with AR to better characterize RV remodelling and determine its prognostic value. For patients with severe AR, because interventions may be the only effective treatment (2, 3), it is necessary to further carefully monitor and actively investigate such patients in the next stage of the study to determine the best timing of the early operation and the prognosis norms.

### Study limitations

First, this is an observational study, which may lead to selection bias. Second, the study had a limited follow-up time (2 years), which may affect the clinical outcomes and analytic results. Third, although this study used a combination of qualitative and quantitative methods to define the severity of AR, the database generally did not include further qualitative indicators. Furthermore, although the study scale was sufficient, the exploratory results need to be further confirmed in future studies. Fourth, although the right heart catheter is the gold standard for evaluating sPAP, the measurement error between the right cardiac catheter and echocardiography needs to be evaluated more carefully, considering that echocardiography is more widely performed. Finally, because the sample size of the baseline RVD was limited, propensity score matching was not performed to better homogenize patient characteristics. In future studies, larger cohorts are needed to confirm RVD outcomes and prognostic effects in patients with AR after TAVR.

## Conclusions

In patients with AR who undergo TAVR, RVD was common and correlated with worsening clinical status. Periprocedural RVD status, whether RVD improvement or the occurrence of new-onset or residual RVD after TAVR, significantly affected the risk-stratified outcomes after TAVR. New-onset RVD was correlated with an increased risk of all-cause death and the composite end point, and normalization of RVD improved clinical outcomes of baseline RVD. These findings suggested the need to include RVD assessment as part of the decision-making and risk assessment strategies for these patients. The next step is to further evaluate appropriate treatment strategies in prospective clinical studies.

### Impact on daily practice

The prognostic value of right ventricular dysfunction (RVD) in patients undergoing transcatheter aortic valve replacement remains unclear. This study showed the clinical implications and prognostic value of RVD after transcatheter aortic valve replacement in patients with pure aortic regurgitation. New-onset RVD was correlated with an increased risk of all-cause death and the composite end point, and normalized RVD improved clinical outcomes of baseline RVD.

## Data Availability

The datasets presented in this study can be found in online repositories. The names of the repository/repositories and accession number(s) can be found in the article/Supplementary Material.
