# Supplementary material for: Prognostic value of right ventricular dysfunction in aortic regurgitation after transcatheter aortic valve replacement
Source: Front Cardiovasc Med. 2024 Aug 30;11:1424116. doi: 10.3389/fcvm.2024.1424116 (PMC11392773; doi:10.3389/fcvm.2024.1424116)
Supplement: Supplementary file 1 [file Table1.pdf]

Supplementary Material

Supplementary Table 1 Univariable and Multivariable Cox Regression Analyses of 2-Year Composite End Point

	Univariate Analysis		Multivariable Analysis	
	HR (95% CI)	*P* Value	HR (95% CI)	*P* Value
Female	0.428 (0.270, 0.678)	0.001	0.554 (0.325, 0.946)	0.030
Diabetes mellitus	2.496 (1.809, 3.444)	< 0.001		
Hypercholesterolaemia	2.480 (1.808, 3.403)	0.002		
Stroke or TIA	2.109 (1.370, 3.124)	< 0.001		
STS score	1.140 (1.090, 1.192)	< 0.001		
Left ventricular ejection fraction	0.956 (0.936, 0.975)	< 0.001		
Left atrial volume index	1.016 (1.004, 1.029)	0.012		
RV–RA gradient	1.029 (1.006, 1.053)	0.014		

TR peak velocity	1.675 (1.354, 2.072)	< 0.001		
New-onset RVD group vs. No RVD group	1.973 (1.274, 3.055)	< 0.001	1.785 (1.013, 3.173)	0.049
Normalized RVD group vs. No RVD group	3.070 (1.973, 4.776)	< 0.001		
Residual RVD group vs. No RVD group	2.991 (1.981, 4.517)	< 0.001		
TAPSE	0.607 (0.446, 0.825)	0.001		

RVD: right ventricular dysfunction; TIA: transient ischemic attack; STS: Society of Thoracic Surgeons; RV: right ventricle; RA: right atrium; TAPSE: tricuspid annular plane systolic excursion; TR: tricuspid regurgitation.

Supplementary Table 2 Univariable and Multivariable Logistic Regression Analyses of No Right Ventricular Dysfunction

	Univariate Analysis		Multivariable Analysis	
	HR (95% CI)	*P* Value	HR (95% CI)	*P* Value
**Preprocedural FAC < 35% as a binary variable**				
Body mass index	0.913(0.855,0.974)	0.006		
Body surface area	0.352(0.140,0.868)	0.025		
STS score	1.073(1.004,1.147)	0.038	1.187(1.029,1.391)	0.025
LVEDV	1.030(1.008,1.053)	0.007	0.702(0.528,0.885)	0.007
LVESV	1.102(1.066,1.140)	< 0.001		
Left ventricular ejection fraction	0.881(0.844,0.918)	< 0.001	0.561(0.357,0.880)	0.012
LA volume index	1.052(1.032,1.074)	< 0.001		
Tricuspid regurgitation ≥ moderate	21.923(12.800,38.742)	< 0.001		

Systolic pulmonary artery pressure	1.167(1.133,1.207)	< 0.001	1.087(1.038,1.141)	0.001
RV–RA gradient	1.092(1.053,1.135)	< 0.001		
TR peak velocity	5.983(3.813,9.695)	< 0.001		
RV basal diameter	0.821(0.781,0.858)	< 0.001	0.880(0.823,0.935)	< 0.001
**Preprocedural FAC < 35% as a continuous variable**				
Body mass index	0.913(0.855,0.974)	0.006		
Body surface area	0.352(0.140,0.868)	0.025		
STS score	1.073(1.004,1.147)	0.038	1.190(1.030,1.398)	0.025
LVEDV	1.030(1.008,1.053)	0.007	0.724(0.541,0.920)	0.016
LVESV	1.102(1.066,1.140)	< 0.001		
Left ventricular ejection fraction	0.881(0.844,0.918)	< 0.001	0.550(0.349,0.863)	0.009

LA volume index	1.052(1.032,1.074)	< 0.001		
Tricuspid regurgitation ≥ moderate	21.923(12.800,38.742)	< 0.001		
Systolic pulmonary artery pressure	1.167(1.133,1.207)	< 0.001	1.086(1.038,1.140)	0.001
RV–RA gradient	1.092(1.053,1.135)	< 0.001		
TR peak velocity	5.983(3.813,9.695)	< 0.001		
RV basal diameter	0.821(0.781,0.858)	< 0.001	0.887(0.830,0.943)	< 0.001
**Preprocedural TAPSE < 1.7 cm as a binary variable**				
Body mass index	0.913(0.855,0.974)	0.006		
Body surface area	0.352(0.140,0.868)	0.025		
STS score	1.073(1.004,1.147)	0.038	1.180(1.026,1.378)	0.027
LVEDV	1.030(1.008,1.053)	0.007	0.684(0.518,0.859)	0.003

LVESV	1.102(1.066,1.140)	< 0.001		
Left ventricular ejection fraction	0.881(0.844,0.918)	< 0.001	0.521(0.332,0.811)	0.004
LA volume index	1.052(1.032,1.074)	< 0.001		
Tricuspid regurgitation ≥ moderate	21.923(12.800,38.742)	< 0.001		
Systolic pulmonary artery pressure	1.167(1.133,1.207)	< 0.001	1.100(1.052,1.154)	< 0.001
RV–RA gradient	1.092(1.053,1.135)	< 0.001		
TR peak velocity	5.983(3.813,9.695)	< 0.001		
RV basal diameter	0.821(0.781,0.858)	< 0.001	0.867(0.812,0.920)	< 0.001
**Preprocedural TAPSE < 1.7 cm as a continuous variable**				
Body mass index	0.913(0.855,0.974)	0.006		
Body surface area	0.352(0.140,0.868)	0.025		

STS score	1.073(1.004,1.147)	0.038	1.179(1.022,1.379)	0.031
LVEDV	1.030(1.008,1.053)	0.007	0.685(0.515,0.865)	0.004
LVESV	1.102(1.066,1.140)	< 0.001		
Left ventricular ejection fraction	0.881(0.844,0.918)	< 0.001	0.509(0.324,0.794)	0.003
LA volume index	1.052(1.032,1.074)	< 0.001		
Tricuspid regurgitation ≥ moderate	21.923(12.800,38.742)	< 0.001		
Systolic pulmonary artery pressure	1.167(1.133,1.207)	< 0.001	1.103(1.054,1.158)	< 0.001
RV–RA gradient	1.092(1.053,1.135)	< 0.001		
TR peak velocity	5.983(3.813,9.695)	< 0.001		
RV basal diameter	0.821(0.781,0.858)	< 0.001	0.869(0.814,0.923)	< 0.001
**Preprocedural S' < 9.5 cm/s as a binary variable**				

Body mass index	0.913(0.855,0.974)	0.006		
Body surface area	0.352(0.140,0.868)	0.025		
STS score	1.073(1.004,1.147)	0.038	1.181(1.027,1.379)	0.026
LVEDV	1.030(1.008,1.053)	0.007	0.680(0.515,0.854)	0.003
LVESV	1.102(1.066,1.140)	< 0.001		
Left ventricular ejection fraction	0.881(0.844,0.918)	< 0.001	0.519(0.331,0.808)	0.004
LA volume index	1.052(1.032,1.074)	< 0.001		
Tricuspid regurgitation ≥ moderate	21.923(12.800,38.742)	< 0.001		
Systolic pulmonary artery pressure	1.167(1.133,1.207)	< 0.001	1.099(1.052,1.154)	< 0.001
RV–RA gradient	1.092(1.053,1.135)	< 0.001		
TR peak velocity	5.983(3.813,9.695)	< 0.001		

RV basal diameter	0.821(0.781,0.858)	< 0.001	0.867(0.812,0.920)	< 0.001
**Preprocedural S' < 9.5 cm/s as a continuous variable**				
Body mass index	0.913(0.855,0.974)	0.006		
Body surface area	0.352(0.140,0.868)	0.025		
STS score	1.073(1.004,1.147)	0.038	1.184(1.029,1.383)	0.025
LVEDV	1.030(1.008,1.053)	0.007	0.681(0.516,0.855)	0.003
LVESV	1.102(1.066,1.140)	< 0.001		
Left ventricular ejection fraction	0.881(0.844,0.918)	< 0.001	0.514(0.327,0.802)	0.004
LA volume index	1.052(1.032,1.074)	< 0.001		
Tricuspid regurgitation ≥ moderate	21.923(12.800,38.742)	< 0.001		
Systolic pulmonary artery pressure	1.167(1.133,1.207)	< 0.001	1.100(1.052,1.154)	< 0.001

RV–RA gradient	1.092(1.053,1.135)	< 0.001		
TR peak velocity	5.983(3.813,9.695)	< 0.001		
RV basal diameter	0.821(0.781,0.858)	< 0.001	0.866(0.811,0.919)	< 0.001

STS: Society of Thoracic Surgeons; LA: left atrium; LVEDV: left ventricular end-diastolic volume; LVESV: left ventricular end-systolic volume; FAC: fraction area change; S’: right ventricular lateral systolic motion using tissue doppler imaging; TAPSE: tricuspid annular plane systolic excursion; RV: right ventricle; RA: right atrium; TR: tricuspid regurgitation.

Supplementary Table 3 Univariable and Multivariable Logistic Regression Analyses of Residual Right Ventricular Dysfunction

	Univariate Analysis		Multivariable Analysis	
	HR (95% CI)	*P* Value	HR (95% CI)	*P* Value
**Preprocedural FAC < 35% as a binary variable**				
Coronary artery disease	0.475(0.241,0.914)	0.021		
Permanent pacemaker implantation	0.204(0.011,1.230)	0.045		
EuroSCORE	0.892(0.801,0.989)	0.023	0.926(0.817,1.044)	0.032
Left ventricular ejection fraction	1.980(1.739,2.022)	0.031		
E/e’	2.702(1.590,3.824)	0.024		
Tricuspid regurgitation ≥ moderate	1.731(0.909,3.320)	0.046		
Systolic pulmonary artery pressure	1.033(0.988,1.080)	0.008	1.238(0.592,2.641)	0.017
**Preprocedural FAC < 35% as a continuous variable**				
Coronary artery disease	0.475(0.241,0.914)	0.021	0.609(0.279,1.313)	0.028

Permanent pacemaker implantation	0.204(0.011,1.230)	0.045		
EuroSCORE	0.892(0.801,0.989)	0.023	0.927(0.819,1.046)	0.032
Left ventricular ejection fraction	1.980(1.739,2.022)	0.031		
E/e’	2.702(1.590,3.824)	0.024		
Tricuspid regurgitation ≥ moderate	1.731(0.909,3.320)	0.046		
Systolic pulmonary artery pressure	1.033(0.988,1.080)	0.008	0.984(0.911,1.062)	0.015
**Preprocedural TAPSE < 1.7 cm as a binary variable**				
Coronary artery disease	0.475(0.241,0.914)	0.021	0.606(0.277,1.305	0.023
Permanent pacemaker implantation	0.204(0.011,1.230)	0.045		
EuroSCORE	0.892(0.801,0.989)	0.023	0.930(0.822,1.049)	0.029
Left ventricular ejection fraction	1.980(1.739,2.022)	0.031		

E/e’	2.702(1.590,3.824)	0.024		
Tricuspid regurgitation ≥ moderate	1.731(0.909,3.320)	0.046		
Systolic pulmonary artery pressure	1.033(0.988,1.080)	0.008	1.242(0.601,2.619)	0.017
**Preprocedural TAPSE < 1.7 cm as a continuous variable**				
Coronary artery disease	0.475(0.241,0.914)	0.021	0.602(0.275,1.299)	0.029
Permanent pacemaker implantation	0.204(0.011,1.230)	0.045		
EuroSCORE	0.892(0.801,0.989)	0.023	0.929(0.821,1.048)	0.042
Left ventricular ejection fraction	1.980(1.739,2.022)	0.031		
E/e’	2.702(1.590,3.824)	0.024		
Tricuspid regurgitation ≥ moderate	1.731(0.909,3.320)	0.046		
Systolic pulmonary artery pressure	1.033(0.988,1.080)	0.008	1.022(0.447,2.339)	0.009
**Preprocedural S’ < 9.5 cm/s as a binary variable**				

Coronary artery disease	0.475(0.241,0.914)	0.021	0.601(0.274,1.294)	0.026
Permanent pacemaker implantation	0.204(0.011,1.230)	0.045		
EuroSCORE	0.892(0.801,0.989)	0.023	0.921(0.812,1.040)	0.028
Left ventricular ejection fraction	1.980(1.739,2.022)	0.031		
E/e’	2.702(1.590,3.824)	0.024		
Tricuspid regurgitation ≥ moderate	1.731(0.909,3.320)	0.046		
Systolic pulmonary artery pressure	1.033(0.988,1.080)	0.008	1.144(1.090,1.406)	0.016
**Preprocedural S’ < 9.5 cm/s as a continuous variable**				
Coronary artery disease	0.475(0.241,0.914)	0.021	0.594(0.271,1.280)	0.028
Permanent pacemaker implantation	0.204(0.011,1.230)	0.045		
EuroSCORE	0.892(0.801,0.989)	0.023	0.923(0.815,1.042)	0.030

Left ventricular ejection fraction	1.980(1.739,2.022)	0.031		
E/e’	2.702(1.590,3.824)	0.024		
Tricuspid regurgitation ≥ moderate	1.731(0.909,3.320)	0.046		
Systolic pulmonary artery pressure	1.033(0.988,1.080)	0.008	1.053(0.950,1.171)	0.013

EuroSCORE: European System for Cardiac Operative Risk Evaluation.
